# Formation of Planar π-Conjugated Sheets in Cocrystals of Bis(iodoethynyl)pyridines and Bipyrimidylalkynes: Cooperative C–H···N Hydrogen Bonds and sp-C–I···N Halogen Bonds

**DOI:** 10.1021/acs.cgd.4c01264

**Published:** 2024-11-07

**Authors:** Lydia B. Lang, Nathan P. Bowling, Eric Bosch

**Affiliations:** † Department of Chemistry and Biochemistry, Missouri State University, 901 South National Avenue, Springfield, Missouri 65897, United States; ‡ Department of Chemistry, University of Wisconsin-Stevens Point, 2101 Fourth Avenue, Stevens Point, Wisconsin 54481, United States

## Abstract

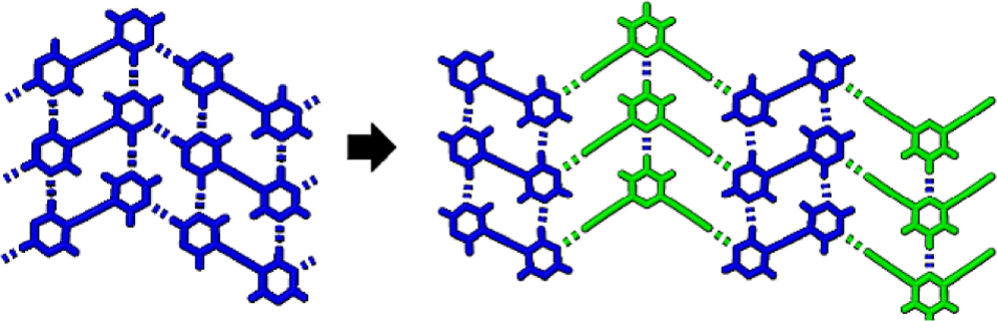

The cocrystallization of the ditopic halogen bond donors 2,5-, 2,6-, 3,5-bis(iodoethynyl)pyridines with the dipyrimidyls 1,2-bis(5-pyrimidyl)ethyne and 1,4-bis(5-pyrimidyl)butadiyne is explored. The cocrystal components have complementary shapes and functional groups so that the noncovalent C–I···N halogen bonding and C–H···N hydrogen bonding interactions are complementary resulting in 1:1 cocrystals with the ditopic halogen bond accepting bipyrimidyls. The cocrystals feature π-stacked planar sheets of alternating bis(iodoalkynes) and bipyrimidyls held together in one direction by I···N halogen bonds and in the roughly orthogonal direction by pyridine–pyridine and pyrimidine–pyrimidine C–H···N hydrogen bonds.

## Abstract 2

Cooperative C–H···N hydrogen bonding and sp-C–I···N halogen bonding in a series of bis(iodoalkynyl)pyridines and bipyrimidines results in the formation of planar conjugated sheets within the cocrystals.

## 1 Introduction

The deliberate cocrystallization, or self-assembly, of organic molecules is now recognized as a viable route to the formation of functional materials in a wide variety of fields.^1^ The cocrystallization, or self-assembly, process is guided by cooperative noncovalent interactions such as π–π stacking and charge-transfer interactions, hydrogen bonding, and, more recently, halogen bonding between the components. The major challenge is therefore to design molecular components that have complementary molecular structures and functional groups so that the cumulative noncovalent interactions guide the formation of the desired structure of the functional material. Self-assembly of organic π-conjugated molecules has applications in electronic and photonic devices where coplanarity is often desirable.^2^ In this simple small molecule study, we probe the application of cooperative halogen bonding and C–H···N hydrogen bonding, along with π–π stacking, to achieve coplanarity within a series of cocrystals. Halogen bonding is recognized as a powerful noncovalent interaction guiding the formation of functional materials including liquid crystals, photo responsive materials and sensors.^3,4^ While once controversial, C–H hydrogen bonding, in particular the C–H···O interaction, is recognized as a significant noncovalent interaction in proteins and nucleic acids.^5^ We have previously demonstrated that C–H hydrogen bonds may play a supportive role in cocrystallization.^6^

**Figure 1:**
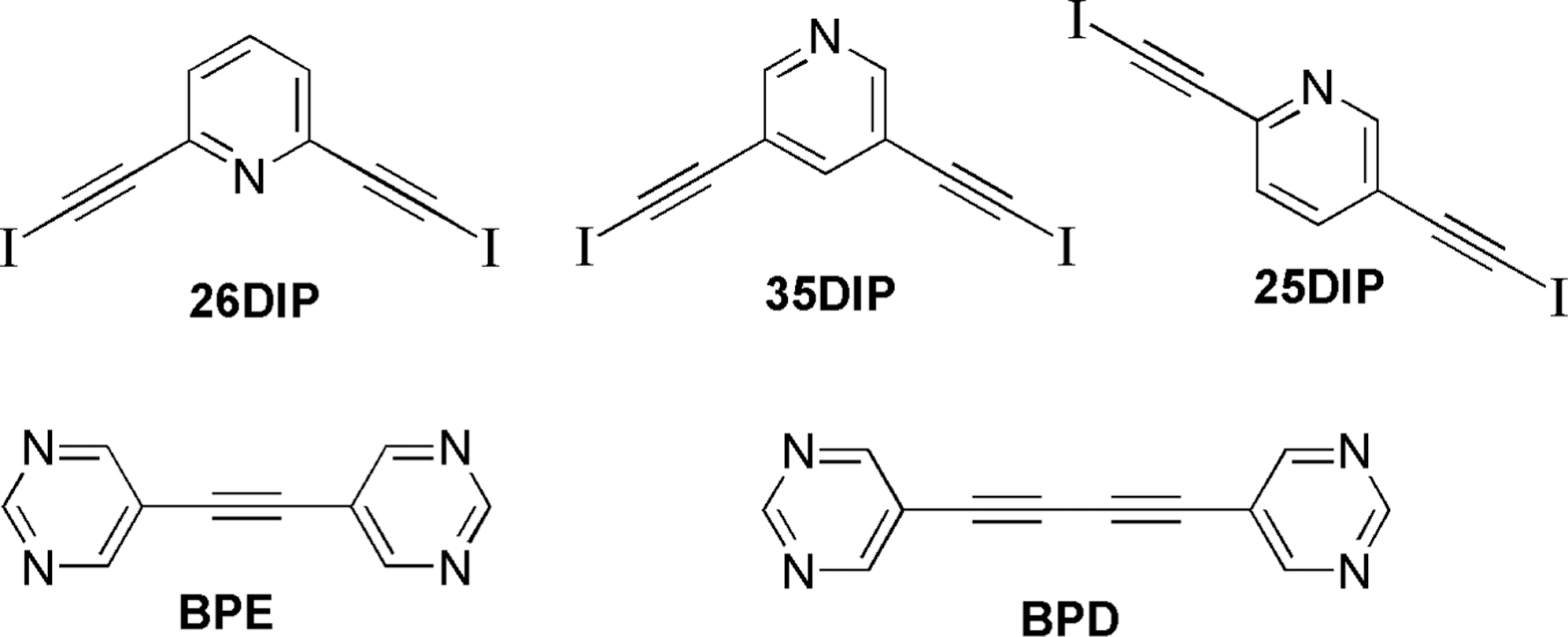
Isomeric bis(iodoalkynyl)pyridine halogen bond donors and the two bipyrimidines used in this study.

The ditopic halogen bond donors in this study are 2,6-, 2,5- and 3,5-bis(iodoethynyl)pyridine, **26DIP**, **25DIP** and **35DIP** respectively. The two bipyrimidines, expected to act as ditopic halogen bond acceptors, are 1,2-bis(5-pyrimidyl)-ethyne and 1,4-bis(5-pyrimidyl)-1,3-butadiyne, **BPE** and **BPD** respectively, as shown in Figure 1. The strategy was based on our knowledge of the crystal structure of each of the bipyrimidines as well as the structures of their cocrystals with diiodotetrafluorobenzenes in which the bipyrimidines were effective ditopic halogen bond acceptors.^7^ In those structures, rows of adjacent bipyrimidines featured two self-complementary C–H···N hydrogen bonds while the linking diiodotetrafluorobenzenes were forced out of planarity with the bipyrimidines due to unfavorable F···F steric and electronic interactions.

## 2 Experimental Section

### 2.1 Synthesis

**BPE**(8) and **BPD**(9) are known compounds. **BPE** was available from earlier studies and **BPD** was synthesized by copper catalyzed homocoupling from 5-ethynyl pyrimidine^10^ as described for the synthesis of the 1,4-dipyridylbutadiyne.^11^ The bis(iodoethynyl)pyridines **26DIP**,^12^**25DIP**^13^ and **35DIP**(14) are known compounds. The formation of these bis(iodoethynyl)pyridines was accomplished by reaction of the corresponding diyne with iodine and sodium hydroxide as described by Aakeröy and co-workers.^15^ 3,5-Diethynylpyridine and 2,6-diethynylpyridine were available from earlier studies^16,17^ and 2,5-diethynylpyridine was prepared as previously described.^18^ All three bis(iodoethynyl)pyridines precipitated as white powders in good yield from the reaction mixtures and were filtered and not purified further as all gave ^1^H and ^13^C NMR spectra identical to published data, most notably the diagnostic ^13^C NMR signal for the sp-C bonded to iodine that appears in the range of 8–16 ppm.

### 2.2 Calculations

The molecules **26DIP**, **25DIP**, and **35DIP** were geometry optimized using the Spartan’20 molecular modeling program with density functional theory (DFT) at the B3LYP/6-311++G** level.^19^ Molecules **BPE** and **BPD** were geometry optimized with the constraint that the pyrimidines were coplanar as this conformation best mimics that observed in cocrystals. The corresponding molecular electrostatic potential energy surfaces were calculated with an isovalue of 0.2 e/au^3^.

The program CrystalExplorer21^20^ was used to calculate the Hirshfeld surface as well as the intermolecular interaction energies within each crystal structure using DFT at the B3LYP level. The fingerprint plots derived from the Hirshfeld surface provide a breakdown of the intermolecular contacts in terms of the atoms within the Hirshfeld surface and atoms outside the surface. The reciprocal interactions, if present, may be included. The interaction energies between pairs of molecules in the crystal is the scaled sum of four physically motivated terms: the classical electrostatic energy (*E*_elec_), the polarization energy (*E*_pol_), the dispersion energy (*E*_dis_), and the exchange-repulsion energy (*E*_rep_) (Mackenzie et al., 2017).^21^

### 2.3 Cocrystal Formation

Equimolar amounts of the two components were weighed and placed in a small screw cap vial. Thus, 7.6 mg (0.02 mmol) of **26DIP** and 3.6 (0.02 mmol) g of **BPE** were weighed and added to a 10 mL screw-cap vial. Absolute ethanol (2 mL) and chloroform (2 mL) were added, and the vial mixed on a vortex mixer. The sealed vial was gently heated with a heat gun and vortexed until the solution was clear. The use of ethanol alone was also explored and while this required larger quantities of ethanol suitable crystals were also obtained. The vial was set aside, and crystals developed over the next 48 h. Crystals were removed from the mother liquor and placed in Paratone oil for X-ray analysis. Both cocrystals with **25DIP** formed using this procedure. Cocrystals **35DIP·BPE** formed upon cooling a warm 1:1 solution of the components in absolute ethanol. Sadly, we were unable to form cocrystals **35DIP·BPD** that were suitable for single crystal X-ray analysis.

### 2.4 Structure Solution

X-ray data for the cocrystals was collected on a Rigaku XtaLAB Synergy diffractometer using Cu Kα radiation (λ = 1.54184 Å) with a HyPix detector. Crystals were immersed in Paratone oil, and a suitable specimen placed on a MiTeGen mount. Crystals were kept at 100.00(1) K during data collection. An analytical numerical absorption correction was applied within CrysAlisPro^22^ using a multifaceted crystal model based on expressions derived by Clark and Reid^23^ for all samples except **26DIP·BPE** where it was Gaussian. The structures were solved in Olex2^24^ with the SHELXT^25^ structure solution program using Intrinsic Phasing and refined with the olex2.refine^26^ refinement package using Gauss–Newton minimization. In all structures hydrogen atoms bound to carbon atoms were observed in the difference Fourier map and were geometrically constrained using the appropriate AFIX commands. The cocrystal **25DIP·BPE** was consistently formed as twinned. Consequently, the structure was solved using the appropriate twin law and one EADP restraint. The data for all five cocrystals is collected in Table S1. Data for **BPE** and **BPD** were previously collected on a Bruker Apex1 diffractometer with Mo Kα radiation and collated in Table S2.

## 3 Results and Discussion

### 3.1 Molecular Electrostatic Potential Calculations

**Figure 2:**
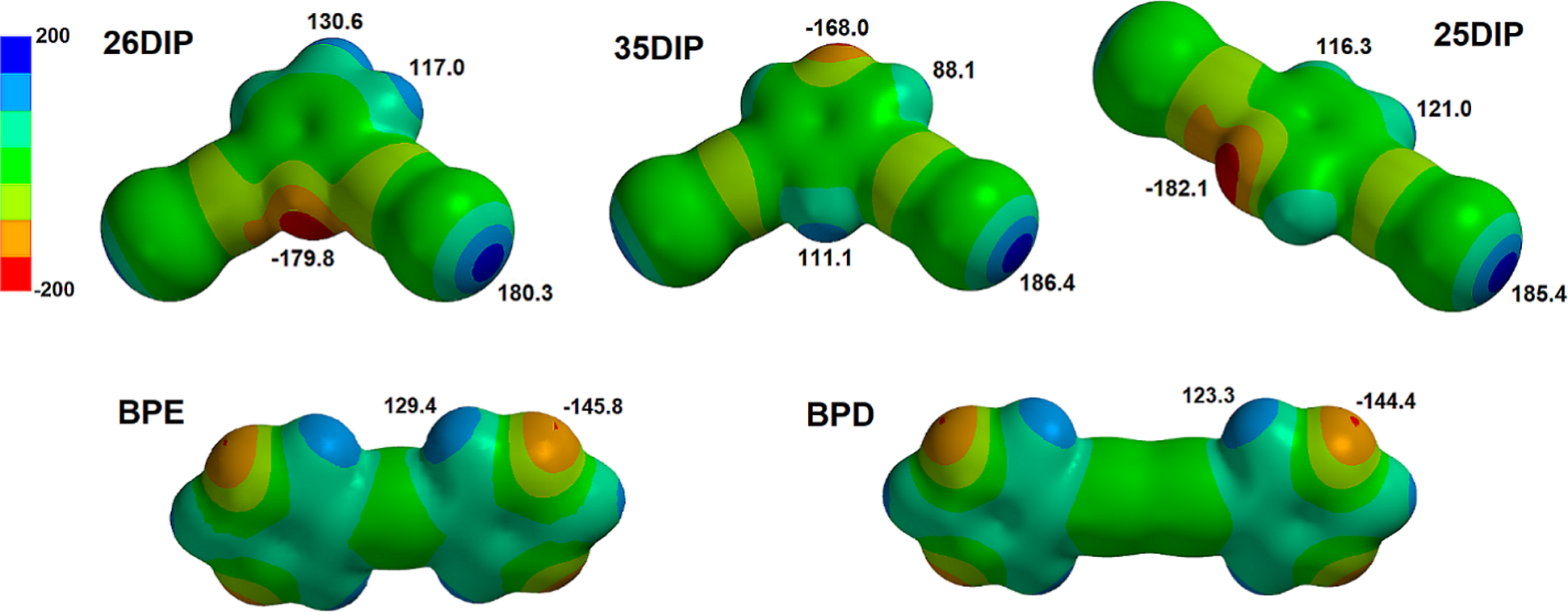
Molecular electrostatic potential surfaces for **26DIP**, **25DIP**, **35DIP**, **BPE** and **BPD** as calculated using Spartan’20.

The molecular electrostatic potentials calculated for the three bis(iodoethynyl)-pyridines and the two bipyrimidines, constrained to the planar conformation, are shown in Figure 2. These plots confirm a strong σ-hole on the iodine atoms with potentials between 180.3 and 186.4 kJ/mol. These values are slightly higher than the 176.4 kJ/mol calculated for iodoperfluorobenzene at the same level confirming the potential of iodoalkynes as good halogen bond donors. Furthermore, the pyridyl N atoms are the center of electron density with electrostatic potentials between −168 and −182 kJ/mol (see Figure 2). It is further noteworthy that the proton para to the N atom has the second highest electrostatic potential in all three compounds, ranging from 130.6 to 111.1 kJ/mol, supporting the potential for intermolecular pyridine–pyridine C–H···N hydrogen bonding. Molecular electrostatic potential calculations for the planar conformation of the bipyrimidines confirm that they are weaker bases than the pyridines with minimum electrostatic potentials on the N atoms around −145 kJ/mol.

### 3.2 X-ray Structures of Bipyrimidines

**Figure 3:**
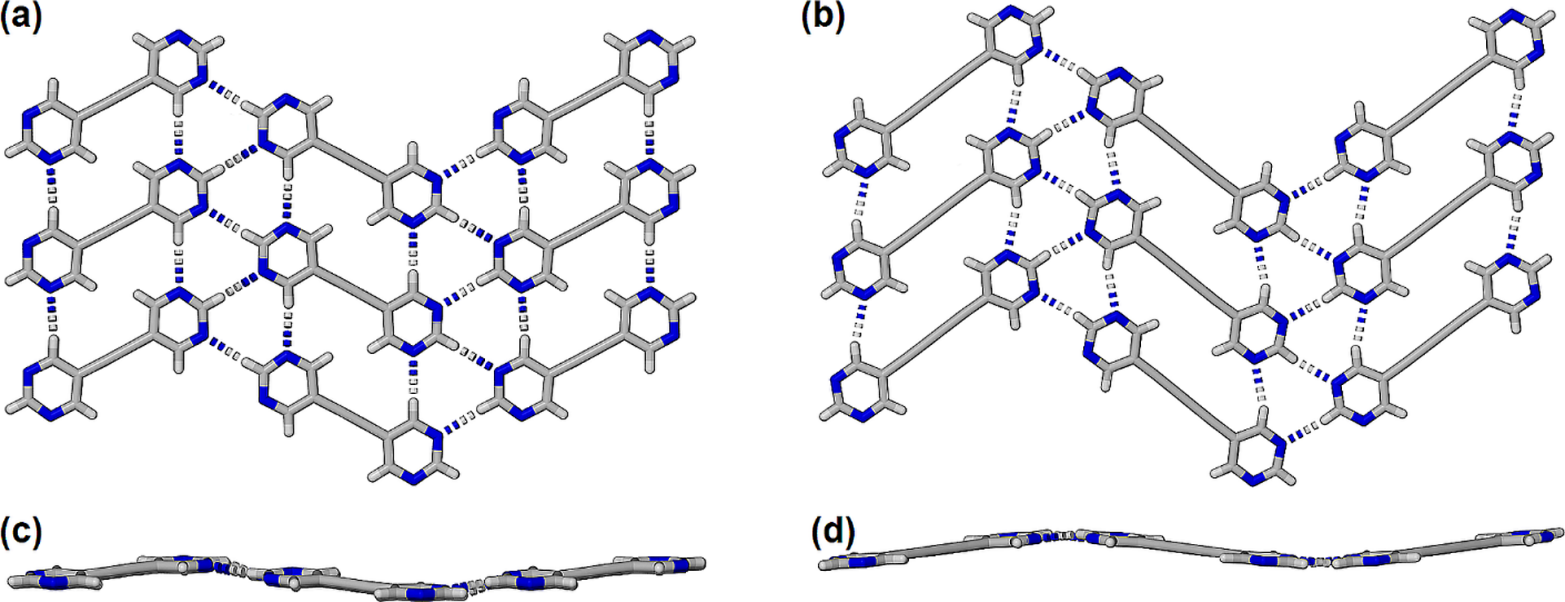
(a) View of the gently corrugated sheets of C–H···N hydrogen bonded molecules **BPE** within the crystal structure. (b) View of the corrugated sheets of C–H···N hydrogen bonded molecules **BPD** within the crystal structure. Views (c,d) correspond to orthogonal views of (a,b) respectively.

The structure of **BPD** has not been deposited in the Cambridge Structural Database^27^ and while the structure of **BPE** at 150 K has recently been published (Meitinger, 2021),^28^ for completeness, we have collected the data for both bipyrimidines at 100 K. Both **BPE** and **BPD** crystallize in the monoclinic space group *P*2_1_/*n* (Table S2). The asymmetric unit of **BPE** has one unique molecule while the asymmetric unit of **BPD** has one-half of one molecule in the asymmetric unit (Figure S1). In Figure 3 a portion of the corrugated sheet of adjacent molecules in each structure is shown along with an orthogonal view. The orthogonal views show the corrugated nature of the 2-dimensional sheets. Within each structure there are two hydrogen bonded rings. The larger parallelogram shaped ring is formed by two self-complementary hydrogen bonds, graph set notation and *R*_2_^2^(16) and *R*_2_^2^(20) respectively, and the smaller triangular ring is formed by three hydrogen bonds with graph set notation *R*_3_^3^(9). In the structure of **BPE** there are 4 unique C–H···N hydrogen bonds with H···N separations between 2.45 and 2.61 Å (Table S3) while in the structure of **BPD** the two unique hydrogen bonds have H···N separations of 2.48 and 2.58 Å (Table S4).

### 3.3 X-ray Stuctures of Cocrystals

**Figure 4:**
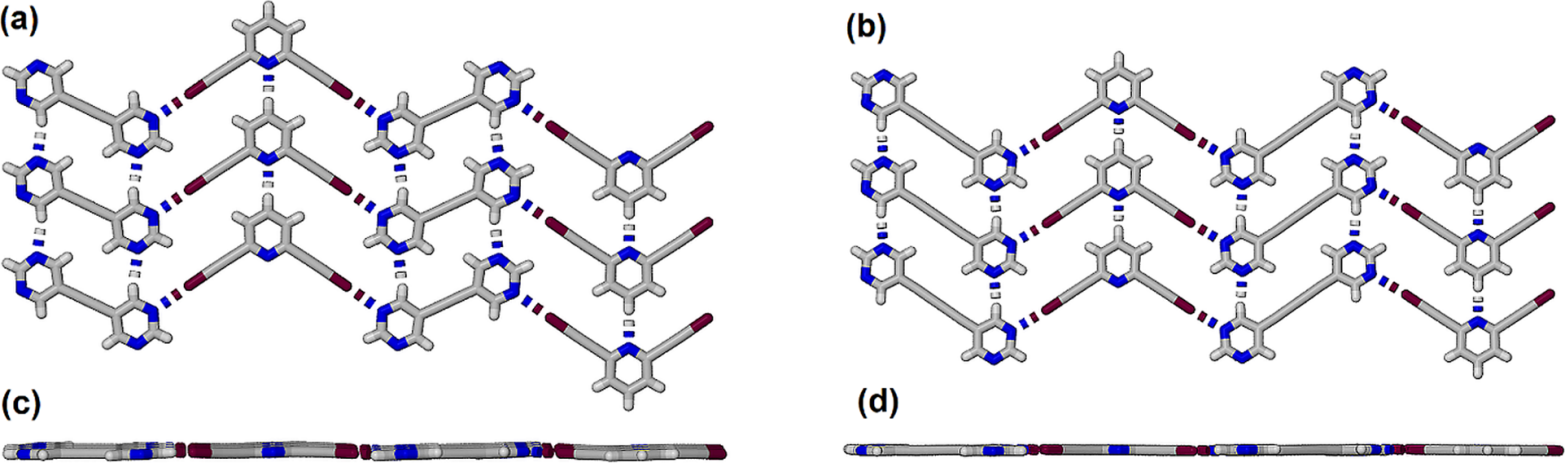
Partial view of the planar sheets formed in the structures cocrystals (a) **26DIP·BPE** and (b) **26DIP·BPD**. (c,d) show the orthogonal view corresponding to (a,b) respectively. Color coded as C gray, N blue and I maroon. Halogen bond shown dashed.

The colorless cocrystals **26DIP·BPE** and **26DIP·BPD** were each formed on cooling a warm 1:1 solution of the components in absolute ethanol. These cocrystals were found to crystallize in the space groups *P*2/*n* and *C*2/*c* respectively (Table S1) with one-half of each component molecule in each respective asymmetric unit (Figure S2). The halogen bonds have I···N separations 2.815(3) and 2.911(2) Å and C–I···N angles 178.95(10) and 177.15(10)° respectively. In each structure the halogen bonds generate seemingly infinite zigzag chains of alternating bis-2,6-(iodoethynyl)pyridine and bipyrimidine molecules. Importantly, the pyrimidines, and bisiodoethynylpyridines, each form C–H···N hydrogen bonded ribbons of molecules orthogonal to the zigzag chains of halogen bonded molecules. This is best illustrated in Figure 4a,b. The rows of bipyrimidines are held together by two self-complementary C–H···N hydrogen bonds while the ribbons of pyridine molecules are also held together by C–H···N hydrogen bonds. In **26DIP·BPE** and **26DIP·BPD** and the pyridine–pyridine H···N distances are 2.397(5) and 2.370(5) Å respectively; and pyrimidine–pyrimidine H···N distances are 2.505(4) and 2.436(4) Å respectively. The planar nature of these 2-dimensional halogen- and hydrogen bonded networks is shown in Figure 4c,d and contrasts the corrugated nature of the sheets formed in **BPE** and **BPD**.

**Figure 5:**
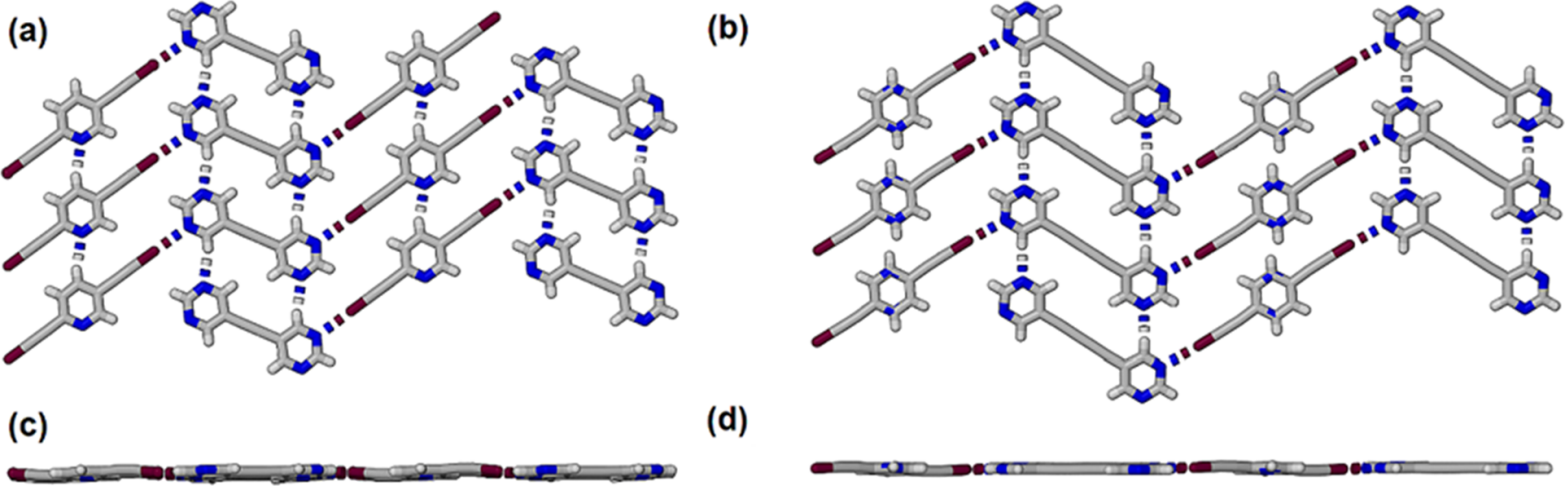
(a) Partial view of the planar sheets formed in the structures cocrystal **25DIP·BPE** and (b) planar sheets in cocrystal **25DIP·BPD**. Note that as the pyridine is disordered the pyridine–pyridine hydrogen bond is not shown. (c,d) correspond to views along each of the planes shown in (a,b) respectively.

The cocrystal **25DIP·BPE** crystallized in the monoclinic space group *C*2/*c* with a unique molecule of each component in the asymmetric unit resulting in two unique halogen bonds. In contrast the cocrystal **25DIP·BPD** crystallized in the monoclinic space group *P*21/*n* with one-half of each molecule in the asymmetric unit (Figure S3). The pyridine was thus disordered between two positions each at 50% occupancy. In both cocrystals planar sheets of molecules were formed with complementary halogen bonded ribbons of alternating bipyrimidyl and bisiodopyridines and self-complementary C–H···N hydrogen bonds between pyridines and between pyrimidines as shown in Figure 5.

**Figure 6:**
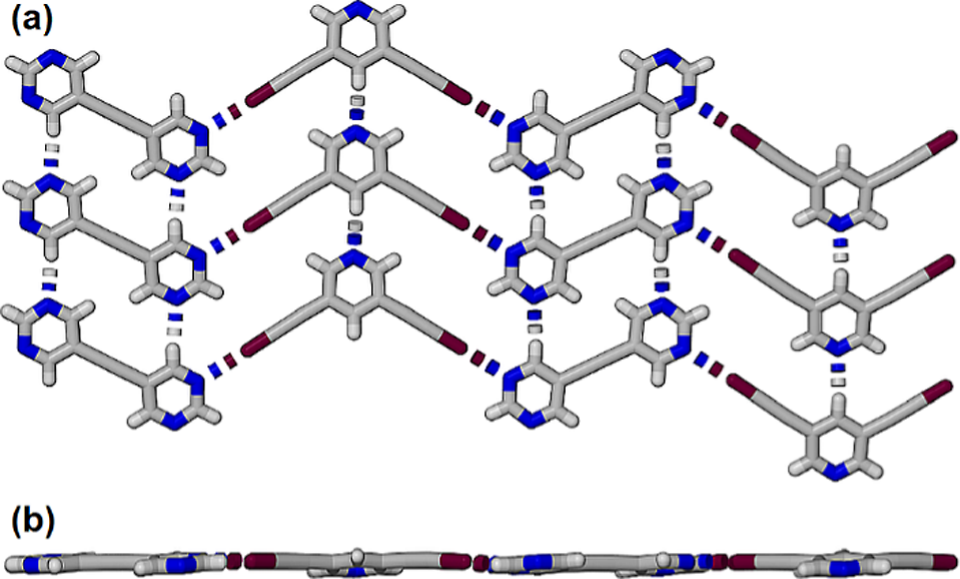
(a) Partial view of the planar sheets formed in the structures cocrystal **35DIP·BPE** and (b) orthogonal view along the plane of molecules shown (a).

The cocrystals **35DIP·BPE** crystallized in the orthorhombic space group *Pbca* with one molecule of each in the asymmetric unit and two unique halogen bonds (Figure S4).. The crystal packing within the cocrystal also comprises of a series of π-stacked planar sheets with molecules in each sheet interacting through cooperative halogen bonding and C–H···N bonding between pyrimidines and between pyridines (Figure 6).

The halogen bond distances in the 5 structures reported here are collated in Table 1. The shortest and longest N···I separation of 2.803 and 2.861 Å correspond to 79.4 and 81.0% of the sum of the van der Waals radii, respectively. The C···I–N angles are essentially linear. The halogen bond distances collated in Table 1 compare favorably to the separations in the two iodoalkyne···pyrimidine cocrystals found in a search of the Cambridge Structural Database.^27^ Those separations between activated iodoalkyne 1-(iodoethynyl)-3,5-dinitrobenzene and each of the activated pyrimidines 5-(furan-2-yl)pyrimidine and 5-(thiophen-2-yl)pyrimidine were 2.794 and 2.825 Å for cocrystals formed.^29^

**Table 1:** Geometric Parameters for Halogen Bonds in the 5 Cocrystals Reported Here

cocrystal	**26DIP·BPE**	**26DIP·BPD**	**25DIP·BPE**^a^	**25DIP·BPD**	**35DIP·BPE**^a^
I···N, Å	2.815(3)	2.811(2)	2.827(6), 2.844(6)	2.803(6)	2.852(3), 2.861(3)
C–I···N, deg	178.95(10)	177.15(10)	177.0(2), 177.2(2)	176.9(3)	178.39(12), 177.79(11)

a In these structures there are two unique iodine atoms.

The data for the C–H···N interactions within the 5 structures reported here are collated in Table 2. For the pyridine···pyridine C–H···N hydrogen bonds the contacts range from 2.370 to 2.523 Å, 86.0–91.7% of the sum of the van der Waals radii with CH···N angles between 157 and 180°. To put these distances in context we searched the Cambridge Structural Database for C–H···N pyridine···pyridine interactions with separation less than or equal to the sum of the van der Waals radii of 2.75 Å. We further constrained the search to close contacts between a pyridine N and the H para to the N in a second pyridine ring and the C–H···N angle between 135 and 180° (Figure S5) to exclude perpendicular interactions. This yielded 1057 hits with 1280 unique pyridine–pyridine interactions. A scatter plot of the H···N distances and C–H···N angles is shown in Figure S6 revealing few interactions with separations below 2.4 Å and 86% of reported interactions above 2.5 Å. Among these, the shortest separation reported is 2.199 Å (80% of the sum of the van der Waals radii) observed in the crystal structure of 2,3,5-trifluoropyridine.^30^

**Table 2:** Geometric Parameters for Pyridine–Pyridine and Pyrimidine–Pyrimidine C–H···N Hydrogen Bonds in the 5 Cocrystals Reported Here

cocrystal	**26DIP·BPE**	**26DIP·BPD**	**25DIP·BPE**	**25DIP·BPD**	**35DIP·BPE**
Pyridine–Pyridine					
H···N, Å	2.397(5)	2.370(5)	2.523(5)	2.460(16)	2.472(5)
C–H···N, deg	180.0(3)	180.0(2)	161.3(8)	176.7(6)	157.7(3)
C···N, Å	3.347(5)	3.320(5)	3.417(5)	3.378(15)	3.370(5)
Pyrimidine–Pyrimidine					
H···N, Å	2.505(4)	2.436(4)	2.574(8), 2.549(8)	2.434(11)	2.459(5), 2.432(5)
C–H···N, deg	167.9(3)	172.0(4)	166.0(10), 163.2(7)	162.6(6)	166.3(3)
C···N, Å	3.440(4)	3.379(4)	3.476(8), 3.458(8)	3.383(11)	3.389(5), 3.371(5)

The H···N separations for the C–H···N pyrimidine···pyrimidine interaction is slightly longer than the pyridine–pyridine interactions and range from 2.434 to 2.574 Å corresponding to 88.5–93.6% of the sum of the van der Waals radii. The C–H···N angles range from 157 to 167°. A similar search for this interaction in the Cambridge Database (see Figure S5b) revealed a total of 283 hits with 346 unique interactions that are plotted in Figure S7. The values reported herein are among the shorter interactions reported perhaps due to the complementarity of the I···N and H···N noncovalent interactions within the structures.

The 1:1 cocrystal structures featuring cooperative halogen bonding and C–H hydrogen bonding reported here augment the similar observations in the crystallization of bis(iodoethynyl)pyrazines from a variety of solvents. Thus, 2,5-bis-iodoethynylpyrazine, when crystallized from acetonitrile, formed planar sheets of molecules within the crystals arranged in a complex tessellation held together by both halogen bonds and C–H···N hydrogen bonds.^31^

### 3.4 Hirshfeld Surface and Intermolecular Energy of Interaction Analysis

**Figure 7:**
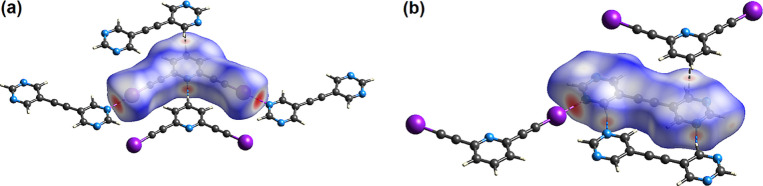
(a) Hirshfeld surface for molecule **26DIP** in cocrystal **26DIP·BPE** and (b) Hirshfeld surface for molecule **BPE** in cocrystal **26DIP·BPE** with *d*_norm_ mapped over the surface. In (a) the two halogen-bonded **BPE** molecules, a p-stacked **BPE** molecule and a hydrogen bonded **26DIP** molecule are shown. In (b) a halogen bonded **26DIP** molecule, a p-stacked **26DIP** molecule and a hydrogen bonded **BPE** molecule are shown. Short contacts shown with dashed lines.

These I···N and H···N close contacts are readily visualized using the Hirshfeld surface plot in which the contacts closer than the sum of the van der Waals radii are colored red. Representative plots shown in Figure 7 correspond to cocrystal **26DIP·BPE** with one view (a) showing close contacts to the Hirshfeld surface of **26DIP** and the second view (b) showing close contacts to the Hirshfeld surface of **BPE**. With respect to the surface of the bis(iodoethynyl) pyridine **26DIP**, the I···N contact corresponds to 6.5% of the surface area while the N–H···C interaction, along with the reciprocal C–H···N interaction, corresponds to 7.5% of the **26DIP** surface area (Figure 7a). The major interactions in terms of surface area of **26DIP** are the C···H and I···H interactions, each with its reciprocal interaction, that cover 36.3 and 28.2% of the surface, respectively. Similar analysis of the surface of the **BPE** molecule reveals that the N···I interaction corresponds to 5.0% of the surface area of the **BPE** molecule while the N···H–C interaction and its reciprocal C–H···N interaction correspond to 20.3% of the **BPE** surface area. The C···H and H···H interactions correspond to 30.7 and 15.6% of the **BPE** surface area, respectively.

**Figure 8:**
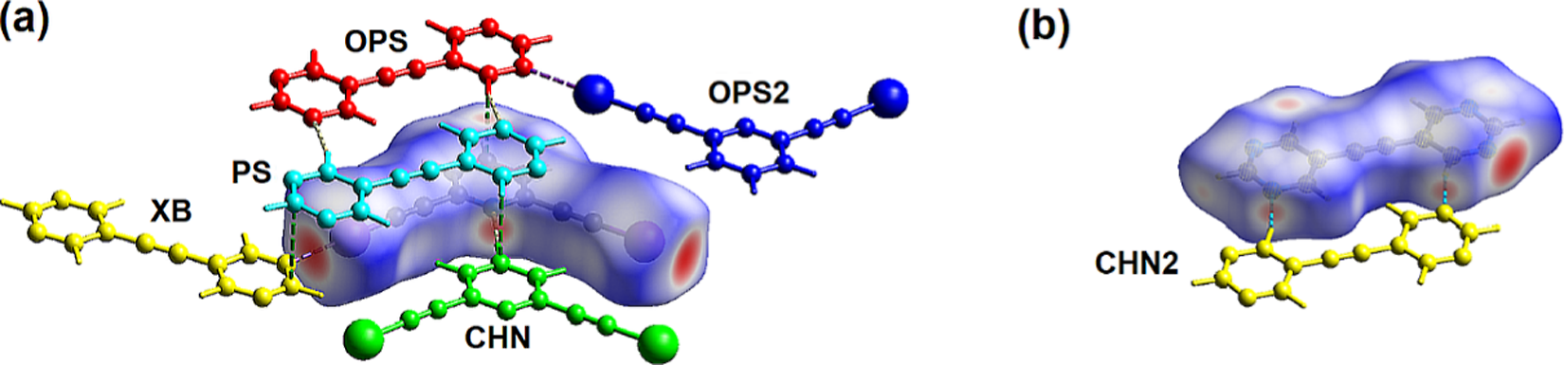
(a) Plot showing molecules within 3.8 Å of **26DIP** in cocrystal **26DIP·BPE** that have significant intermolecular energy of attraction. (b) Plot showing molecules within 3.8 Å of **BPE** in cocrystal **26DIP·BPE** that have significant intermolecular energy of attraction not shown in (a).

To put the surface atom-to-atom contacts in perspective, the intermolecular energy between molecules within the crystal structure **26DIP·BPE** was calculated using CrystalExplorer21.^20^ Thus, the intermolecular energy of interaction for molecules with 3.8 Å of each component in the cocrystal **26DIP·BPE** was calculated and the unique molecules with energy of interaction greater than −10 kJ/mol included in Figure 8. This total energy of interaction is the scaled sum of electrostatic, polarization, dispersion, and repulsive contributions as collated in Table 3.

**Table 3:** Interaction Energies of 5 Unique Molecules with **26DIP** and One Molecule with **BPE** within Cocrystal **26DIP·BPE** as Shown in Figure 8a,b^a^

color, code	*R*	*E*_ele_	*E*_pol_	*E*_dis_	*E*_rep_	*E*_tot_
yellow, **XB**	11.67	–60.2	–6.2	–9.7	89.3	–21.4
green, **CHN**	6.15	–23.6	–5.2	–23.0	34.3	–27.7
light blue, **PS**	4.88	–19.6	–1.6	–38.4	45.9	–26.9
red, **OPS**	6.23	–9.5	–1.1	–25.1	24.1	–17.8
dark blue, **OPS2**	9.32	–7.5	–0.6	–20.6	22.7	–12.2
yellow, **CHN2**	6.16	–23.1	–5.1	–18.3	34.3	–23.0

a *R* is the distance between molecular centroids in Å and all energies are given in kJ/mol. Scale factors for benchmarked energy models see Mackenzie (2017).

Molecules within 3.8 Å of **26DIP** in cocrystal **26DIP·BPE** with significant energy of interaction are shown color coded in Figure 8a. The halogen bonded molecule colored yellow and labeled **XB** in Figure 8a has intermolecular interaction energy of −21.4 kJ/mol. This halogen bonded molecule has a small, relatively localized halogen bond contact area (6.5% of the surface area of **DIP**), thus the electrostatic contribution dominates the interaction energy. In contrast the C–H···N hydrogen bonded molecule colored green and labeled **CHN** in Figure 8a, also has C−H···I close contacts and the strongest intermolecular energy of interaction of −27.7 kJ/mol, has larger surface area of contact and similar electrostatic and dispersion contributions. The π-stacked molecule, light blue/**PS** in Figure 8a, reasonably has a high dispersion contribution with an overall intermolecular energy of interaction of −26.9 kJ/mol. The offset π-stacked **BPE** molecule, red/**OPS** in Figure 8a, and offset **26DIP** molecule, dark blue/**OPS2**, have overall intermolecular energies of interaction of −17.8 and −12.2 kJ/mol, respectively. The intermolecular energy analysis is completed by calculating the energy of interaction between adjacent bipyrimidines **BPE** shown in Figure 8b. The yellow molecule **CHN2** in Figure 8b with self-complementary C–H···N interactions have similar electrostatic and dispersion components for overall intermolecular energy of interaction of −23.0 kJ/mol.

**Figure 9:**
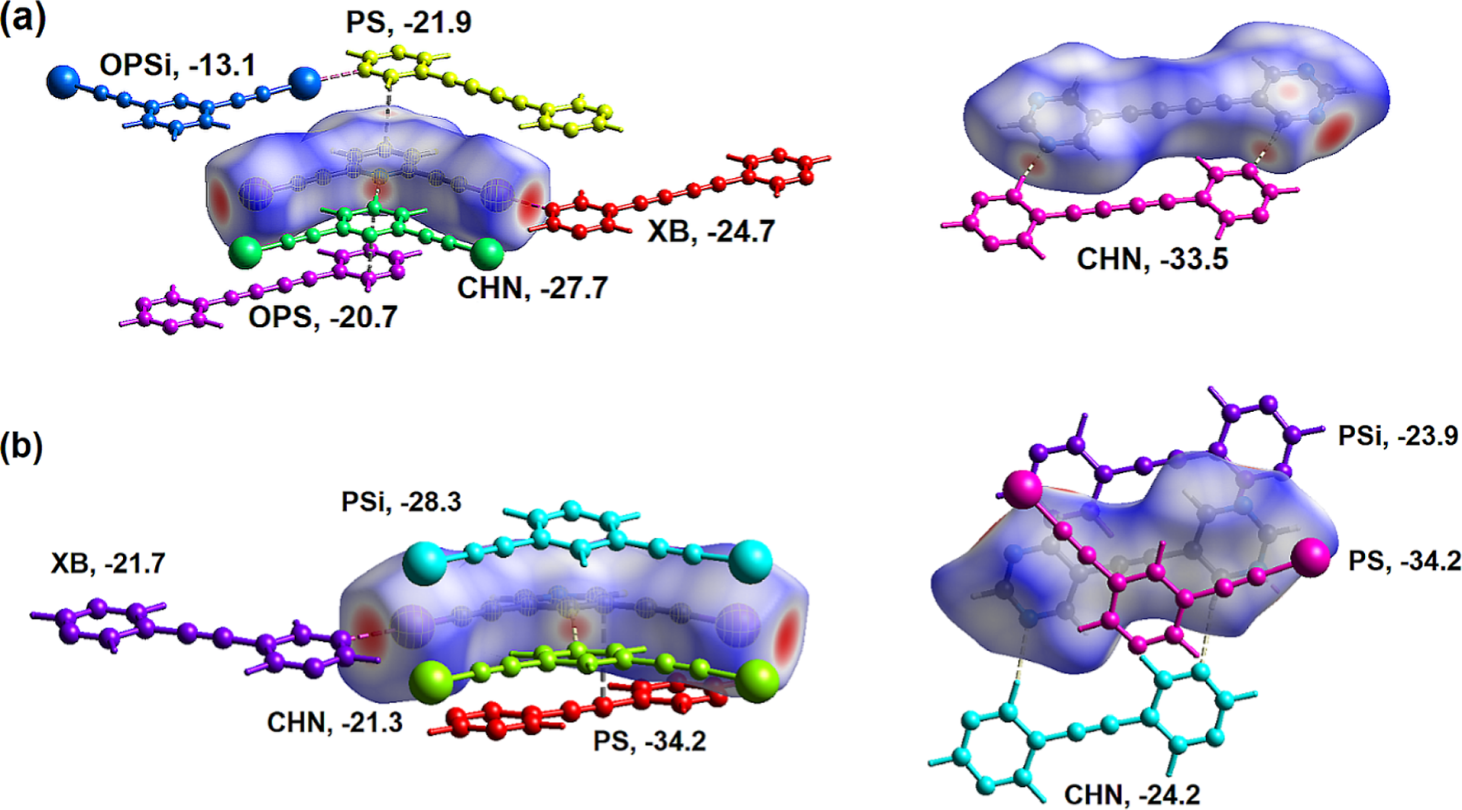
Annotated plots of the intermolecular energies of interaction between neighboring molecules in the structures of cocrystals (a) **26DIP·BPD** and (b) **35DIP·BPE**. The major interactions are annotated as **XB** (halogen bonded); **CHN** (hydrogen bonded); **PS** (π-stacked); **OPS** and **OPSi** (offset π-stacked).

Results for similar analysis of cocrystals **26DIP·BPD** and **35DIP·BPE** are shown annotated in Figure 9.

Across the three sets of intermolecular interaction energy correlated mainly to the site-specific halogen bond, molecules labeled **XB** in Figures 8 and 9, are similar being −21.7, −24.7, and −21.3 kJ/mol. The pyrimidine–pyrimidine C–H···N hydrogen bonded **BPE** molecules have interaction energies of −23.0 and −24.2 kJ/mol while the longer **BPD** molecule (cocrystal **26DIP·BPD**) has a significantly larger pyrimidine–pyrimidine energy of interaction of −33.5 kJ/mol. This is presumably due to increased surface–surface contact area including a weak C–H···π interaction. The π-stacked interactions vary widely as expected due to the offset nature of most of these interactions. The highest intermolecular energy of interaction related to π-stacked molecules, −34.2 kJ/mol, corresponds to those molecules with maximum overlap where the center of gravity of the π-stacked **35DIP** and **BPE** molecules is 3.62 Å (Figure 9b).

## 4 Conclusions

Halogen bonding and cooperative C–H hydrogen bonding along with the ability of the components to π-stack were the guiding principles in the formation of these cocrystals. Cooperative halogen and hydrogen bonding accomplish the goal of formation of planar two-dimensional sheets with all 1:1 cocrystals featuring alternating molecular components showcasing the shape and molecular compatibility of the bis(iodoethynyl)pyridine and bipyrimidylalkyne components. Interestingly the energies of interaction between: (a) halogen bonded molecules, (b) hydrogen bonded bis(iodoethynyl)pyridine molecules; (c) hydrogen bonded bipyrimidyl molecules and, (d) π-stacked molecules are similar even though there is no control over the π-stacking with this set of molecules. Further development of this strategy will require inclusion of molecular moieties with potential optical or electronic application that are designed to π-stack predictably.

## Supporting Information Available

Tabulated crystallographic details for cocrystals **26DIP·BPE**, **26DIP·BPD**, **25DIP·BPE**, **25DIP·BPD** and **35DIP·BPE** as well as structures **BPE** and **BPD**. Tabulated hydrogen bond geometry for structures **BPE** and **BPD**. Asymmetric unit figures for structures **BPE** and **BPD** and for each cocrystal **26DIP·BPE**, **26DIP·BPD**, **25DIP·BPE**, **25DIP·BPD** and **35DIP·BPE**. Criteria for CCDC database searches along with scattergram plots of C–H···N interactions in pyridine and pyrimidine structures from the database (PDF)

## References

[ref1] Sun L.; Zhu W.; Zhang X.; Li L.; Dong H.; Hu W. Creating Organic Functional Materials beyond Chemical Bond Synthesis by Organic Cocrystal Engineering. J. Am. Chem. Soc. 2021, 143, 19243–19256. 10.1021/jacs.1c07678. 34730972

[ref2] Liu H. Y.; Li Y. C.; Wang X. D. Recent advances in organic donor–acceptor cocrystals: design, synthetic approaches, and optical applications. CrystEngComm 2023, 25, 3126–3141. 10.1039/D3CE00146F.

[ref3] Kampes R.; Zechel S.; Hager M. D.; Schubert U. S. Halogen bonding in polymer science: towards new smart materials. Chem. Sci. 2021, 12, 9275–9286. 10.1039/D1SC02608A. 34349897 PMC8278954

[ref4] Zheng J.; Suwardi A.; Wong C. J. E.; Loh X. J.; Li Z. Halogen bonding regulated functional nanomaterials. Nanoscale Adv. 2021, 3, 6342–6357. 10.1039/D1NA00485A. 36133496 PMC9419782

[ref5] Derewenda Z. S. C-H Groups as Donors in Hydrogen Bonds: A Historical Overview and Occurrence in Proteins and Nucleic Acids. Int. J. Mol. Sci. 2023, 24, 13165 10.3390/ijms241713165. 37685972 PMC10488043

[ref6] Bosch E.; Bowling N. P. Cooperative Strong Charge-Assisted N–H···O Hydrogen Bonding and Weaker Nonconventional C–H···N Hydrogen Bonding in the Formation of Extended Hydrogen-Bonded Networks with 2,3,5,6-Tetrafluorobenzoic Acid. Cryst. Growth Des. 2020, 20, 1565–1571. 10.1021/acs.cgd.9b01277.

[ref7] Nwachukwu C. I.; Patton L. J.; Bowling N. P.; Bosch E. Ditopic halogen bonding with bipyrimidines and activated pyrimidines. Acta Crystallogr., Sect. C: Struct. Chem. 2020, 76, 458–467. 10.1107/S2053229620005082. 32367827

[ref8] Georgiev I.; Bosch E.; Barnes C. L.; Draganjac M. The Quest for Chain-Link Hydrogen-Bonded Capsules: Self-Assembly of C-Methyl Calix[4]resorcinarene with 1,2-Bis(5‘-pyrimidyl)ethyne. Cryst. Growth Des. 2004, 4, 235–239. 10.1021/cg034108c.

[ref9] Kim W. H.; Kodali N. B.; Kumar J.; Tripathy S. K. A Novel, Soluble Poly(diacetylene) Containing an Aromatic Substituent. Macromolecules 1994, 27, 1819–1824. 10.1021/ma00085a023.

[ref10] Momose A. A.; Bosch E. Serendipity in the Crystallization of a Series of C-Alkylcalix[4]resorcinarenes from Alcoholic Solvents. Cryst. Growth Des. 2010, 10 (9), 4043–4049. 10.1021/cg100737c.

[ref11] Shi Y.; Yue R.; Zhang Y.; Lv S.; Bai L.; Zhang C.; Wen X. Cu powder/n-butylamine: An effective catalytic system for homo- and crosscoupling of terminal alkynes under ambient conditions. Catal. Commun. 2019, 124, 103–107. 10.1016/j.catcom.2019.02.001.

[ref12] Barry D. E.; Hawes C. S.; Blasco S.; Gunnlaugsson T. Structure Direction, Solvent Effects, and Anion Influences in Halogen-Bonded Adducts of 2,6 Bis(iodoethynyl)pyridine. Cryst. Growth Des. 2016, 16, 5194–5205. 10.1021/acs.cgd.6b00766.

[ref13] Kosaka Y.; Yamamoto H. M.; Nakao A.; Kato R. Multicomponent molecular conductors with supramolecular assemblies prepared from neutral iodine-bearing pBIB (p-bis(iodoethynyl)benzene) and derivatives. Bull. Chem. Soc. Jpn. 2006, 79, 1148–1154. 10.1246/bcsj.79.1148.

[ref14] Brotherton W. S.; Clark R. J.; Zhu L. Synthesis of 5-Iodo-1,4-disubstituted-1,2,3-triazoles Mediated by in Situ Generated Copper(I) Catalyst and Electrophilic Triiodide Ion. J. Org. Chem. 2012, 77, 6443–6455. 10.1021/jo300841c. 22780866

[ref15] Wijethunga T. K.; D̵aković M.; Desper J.; Aakeröy C. B. A new tecton with parallel halogen-bond donors: a path to supramolecular rectangles. Acta Crystallogr., Sect. B: Struct. Sci., Cryst. Eng. Mater. 2017, 73, 163–167. 10.1107/S2052520616016450. 28362278

[ref16] Bosch E.; Barnes C. L. Synthesis and Crystallographic Characterization of a Novel Platinocycle. Organometallics 2000, 19, 5522–5524. 10.1021/om0004904.

[ref17] Bosch E. Role of *sp*-C−H···N Hydrogen Bonding in Crystal Engineering. (2010). Cryst. Growth Des. 2010, 10, 3808–3813. 10.1021/cg100707y.

[ref18] Bunten K. A.; Kakkar A. K. Synthesis of pyridine/pyridinium-based alkynyl monomers, oligomers and polymers: enhancing conjugation by pyridine N-quaternization. J. Mater. Chem. 1995, 5, 2041–2043. 10.1039/jm9950502041.

[ref19] Wavefunction. Spartan’20 Version 1.1.4; Wavefunction Inc.: Irvine, CA, USA, 2022.

[ref20] Spackman P. R.; Turner M. J.; McKinnon J. J.; Wolff S. K.; Grimwood D. J.; Jayatilaka D.; Spackman M. A. *CrystalExplorer*: a program for Hirshfeld surface analysis, visualization and quantitative analysis of molecular crystals. J. Appl. Crystallogr. 2021, 54 (3), 1006–1011. 10.1107/S1600576721002910. 34188619 PMC8202033

[ref21] Mackenzie C. F.; Spackman P. R.; Jayatilaka D.; Spackman M. A. CrystalExplorer model energies and energy frameworks: extension to metal coordination compounds, organic salts, solvates and open-shell systems. IUCrJ 2017, 4, 575–587. 10.1107/S205225251700848X. PMC5600021 28932404

[ref22] Rigaku. CrysAlis PRO; Rigaku Oxford Diffraction: Houston, TX, USA, 2022.

[ref23] Clark R. C.; Reid J. S. The analytical calculation of absorption in multifaceted crystals. Acta Crystallogr., Sect. A: Found. Crystallogr. 1995, 51, 887–897. 10.1107/S0108767395007367.

[ref24] Dolomanov O. V.; Bourhis L. J.; Gildea R. J.; Howard J. A. K.; Puschmann H. OLEX2: a complete structure solution, refinement, and analysis program. J. Appl. Crystallogr. 2009, 42, 339–341. 10.1107/S0021889808042726.

[ref25] Sheldrick G. M. SHELXT - Integrated space-group and crystal-structure determination. Acta Crystallogr., Sect. A: Found. Adv. 2015, 71, 3–8. 10.1107/s2053273314026370. 25537383 PMC4283466

[ref26] Bourhis L. J.; Dolomanov O. V.; Gildea R. J.; Howard J. A. K.; Puschmann H. The anatomy of a comprehensive constrained, restrained refinement program for the modern computing environment – Olex2 dissected. Acta Crystallogr., Sect. A: Found. Adv. 2015, 71, 59–75. 10.1107/S2053273314022207. 25537389 PMC4283469

[ref27] Groom C. R.; Bruno I. J.; Lightfoot M. P.; Ward S. C. The Cambridge Structural Database. Acta Crystallogr., Sect. B: Struct. Sci., Cryst. Eng. Mater. 2016, 72, 171–179. 10.1107/S2052520616003954. PMC4822653 27048719

[ref28] Meitinger N.; Mengele A. K.; Nauroozi D.; Rau S. Pyrimidine-Substituted Hexaarylbenzenes as Versatile Building Blocks for N–Doped Organic Materials. Org. Mater. 2021, 03, 295–302. 10.1055/a-1482-6190.

[ref29] Hardin A. E. S.; Ellington T. L.; Nguyen S. T.; Rheingold A. L.; Tschumper G. S.; Watkins D. L.; Hammer N. I. A Raman Spectroscopic and computational study of new aromatic pyrimidine-based halogen bond acceptors. Inorganics 2019, 7, 119 10.3390/inorganics7100119.

[ref30] Vasylyeva V.; Shishkin O. V.; Maleev A. V.; Merz K. Crystal Structures of Fluorinated Pyridines from Geometrical and Energetic Perspectives. Cryst. Growth Des. 2012, 12, 1032–1039. 10.1021/cg201623e.

[ref31] Ng C. F.; Chow H. F.; Mak T. C. W. Organic molecular tessellations and intertwined double helices assembled by halogen bonding. CrystEngComm 2019, 21, 1130–1136. 10.1039/C8CE02133C.

