# Supplementary material for: Formation of Planar π-Conjugated Sheets in Cocrystals of Bis(iodoethynyl)pyridines and Bipyrimidylalkynes: Cooperative C–H···N Hydrogen Bonds and sp-C–I···N Halogen Bonds
Source: Cryst Growth Des. 2024 Nov 7;24(22):9727–34. doi: 10.1021/acs.cgd.4c01264 (PMC11583210; doi:10.1021/acs.cgd.4c01264)
Supplement: Supplementary file 1 — cg4c01264_si_001.pdf [file cg4c01264_si_001.pdf]

Supporting Information

Formation of planar π-conjugated sheets in cocrystals of bis(iodoethynyl)pyridines and bipyrimidylalkynes: Cooperative C-H---N hydrogen bonds and *sp*-C-I---N halogen bonds

Lydia B. Lang[1] and Nathan P. Bowling[2] and Eric Bosch[1*],

[1]Department of Chemistry and Biochemistry, Missouri State University, 901 South National Avenue, Springfield, Missouri, 65897, USA. [2]Department of Chemistry, University of Wisconsin-Stevens Point, 2101 Fourth Avenue, Stevens Point, WI, 54481, USA.

Correspondence email: ericbosch@missouristate.edu

Table of Contents

Table S1. Crystallographic details for cocrystals ………………………………………… 2

Table S2. Crystallographic details for **BPE, BPD**………………………………………. 3

Figure S1. Assymetric unit of (a) **BPE** and (b) **BPD**. ……………………………………... 4

Table S3. Hydrogen-bond geometry in the structure of **BPE**………………………………… 4

Table S4. Hydrogen-bond geometry in the structure of **BPD**………………………………… 4

Figure S2. Labelled asymmetric unit of (a) **26DIP•BPE** and (b) **26DIP•BPD**……………… 4

Figure S3. Labelled asymmetric unit of (a) **25DIP•BPE** and (b) **25DIP•BPD**……………… 5

Figure S4. Labelled asymmetric unit of **35DIP•BPE**………………………………………… 5

Figure S5. Criteria for CCDC database searches H---N distances……………………………. 5

Figure S6. Scattergram plot of C-H---N hydrogen bonds in pyridine structures ……………… 6

Figure S7. Scattergram plot of C-H---N hydrogen bonds in pyrimidine structures…………..… 6

Table S5. Separation distances for C-H---I and C-H---π interactions………………………….. 7

Table S1. Crystallographic details for cocrystals **26DIP•BPE**, **26DIP•BPD, 25DIP•BPE**, **25DIP•BPD** & **35DIP•BPE**

Experiments were carried out with Cu *K*α radiation at 100K with a XtaLAB Synergy, Dualflex, HyPix diffractometer. Absorption correction using *CrysalisPRO* (Rigaku, 2022). H atom parameters were constrained.

	**26DIP.BPE**	**26DIP.BPD**	**25DIP.BPE**	**25DIP.BPD**	**35DIP.BPE**
Crystal data					
Chemical formula	$C_{10}H_6N_4 \cdot C_9H_3I_2N$	$C_{12}H_6N_4 \cdot C_9H_3I_2N$	$C_{10}H_6N_4 \cdot C_9H_3I_2N$	$C_{12}H_6N_4 \cdot C_9H_3I_2N$	$C_{10}H_6N_4 \cdot C_9H_3I_2N$
M_r	561.13	585.15	561.13	585.15	561.13
Crystal system, space group	Monoclinic, *P*2/*n*	Monoclinic, *C*2/*c*	Monoclinic, *Cc*	Monoclinic, $P2_1/n$	Orthorhombic,*Pbca*
a, *b*, *c* (Å)	9.9637 (1), 6.1520(1), 15.0151 (2)	19.2650 (2), 6.1007 (1), 17.7348 (2)	17.1041 (2), 6.1673, 18.3844 (2)	10.5649 (1), 6.1225 (1), 15.3131 (2)	12.8214 (2), 6.0899 (1), 47.1412 (5)
α, β, γ (°)	90, 90.246 (1), 90	90, 109.168 (1), 90	90, 109.707 (1), 90	90, 94.273 (1), 90	90, 90, 90
V ($Å^3$)	920.37 (2)	1968.81 (5)	1825.71 (3)	987.75 (2)	3680.83 (9)
Z	2	4	4	2	8
μ (mm^{-1})	26.95	25.23	27.17	25.14	26.95
Crystal size (mm)	0.14 × 0.1 × 0.05	0.18 × 0.06 × 0.03	0.19 × 0.1 × 0.03	0.2 × 0.05 × 0.02	0.28 × 0.13 × 0.08
Data collection					
Absorption correction	Gaussian	Analytical	Analytical	Analytical	Analytical
T_{min}, T_{max}	0.121, 0.440	0.068, 0.493	0.051, 0.515	0.203, 0.806	0.010, 0.252
No. of measured, independent and observed [$I \geq 2u(I)$] reflections	8966, 1885, 1805	15434, 1970, 1912	17885, 3344, 3291	9306, 2128, 2070	36963, 3768, 3584
R_{int}	0.037	0.042	0.037	0.042	0.068
$(\sin θ/λ)_{max}$ ($Å^{-1}$)	0.633	0.633	0.633	0.638	0.633
Refinement					
$R[F^2 > 2σ(F^2)]$, $wR(F^2)$, *S*	0.022, 0.060, 1.03	0.025, 0.069, 1.04	0.027, 0.076, 1.04	0.046, 0.105, 1.00	0.033, 0.086, 1.05
No. of reflections	1885	1970	3344	2128	3768
No. of parameters	119	128	236	127	236
No. of restraints	0	0	2	0	0
$Δρ_{max}$, $Δρ_{min}$ (e $Å^{-3}$)	0.55, −0.84	1.13, −1.09	0.57, −0.94	1.67, −1.03	0.89, -2.04
CCDC deposition number	2380561	2380562	2380563	2380564	2380565

Table S2. Crystallographic data for **BPE** and **BPD**.

Structure	**BPE**	**BPD**
Crystal data		
Chemical formula	$C_{10}H_6N_4$	$C_{12}H_6N_4$
M_r	182.19	206.21
Crystal system, space group	Monoclinic, $P2_1/n$	Monoclinic, $P2_1/n$
Temperature (K)	100	100
a, *b*, *c* (Å)	6.1099 (5), 22.4623 (18), 6.9229 (6)	3.736 (3), 25.200 (18), 5.201 (4)
β (°)	113.812 (1)	94.647 (10)
V (Å^3)	869.24 (12)	488.1 (6)
Z	4	2
Radiation type	Mo Kα	Mo Kα
μ (mm^{-1})	0.09	0.09
Crystal size (mm)	0.42 × 0.29 × 0.14	0.16 × 0.14 × 0.10
Data collection		
Diffractometer	Bruker *APEX*-I CCD	Bruker *APEX*-I CCD
Absorption correction	Multi-scan *SADABS* V2008/1 (Bruker AXS)	Multi-scan *SADABS* V2014/5 (Bruker AXS)
T_{min}, T_{max}	0.678, 0.746	0.461, 0.746
No. of measured, independent and observed [$I > 2σ(I)$] reflections	13632, 2930, 2227	3935, 1085, 793
R_{int}	0.028	0.066
(sin θ/λ)$_{max}$ (Å^{-1})	0.752	0.649
Refinement		
$R[F^2 > 2σ(F^2)]$, $wR(F^2)$, S	0.044, 0.133, 1.08	0.096, 0.250, 1.14
No. of reflections	2930	1085
No. of parameters	127	73
H-atom treatment	H-atom parameters constrained	H-atom parameters constrained
Δρ$_{max}$, Δρ$_{min}$ (e Å^{-3})	0.38, −0.20	0.39, −0.33
CCDC Deposition Number	2380448	2380449

Computer programs: *SHELXT* 2018/2 (Sheldrick, 2018), *SHELXL2019*/2 (Sheldrick, 2019).

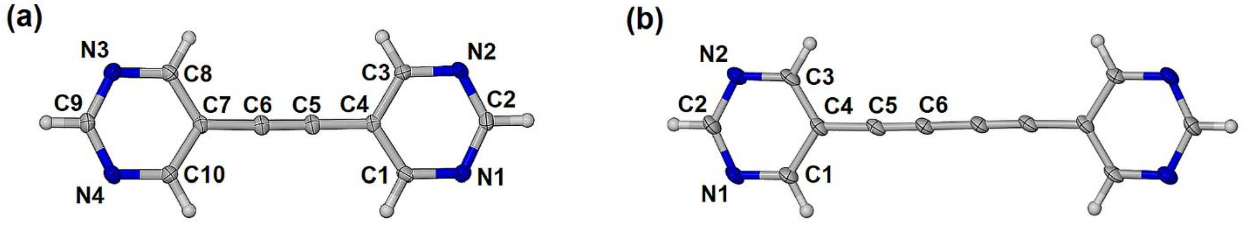

Figure S1. Asymmetric unit of (a) **BPE** and (b) **BPD**.

Table S3. Hydrogen-bond geometry in the structure of **BPE**. (Å, º)

D—H···*A*	*D*—H	H···*A*	*D*···*A*	*D*—H···*A*
C1—H1···N2^{i}	0.95	2.47	3.4050 (13)	167
C8—H8···N4^{ii}	0.95	2.56	3.4544 (13)	157
C2—H2···N3^{iii}	0.95	2.61	3.5172 (13)	160
C9—H9···N1^{iv}	0.95	2.45	3.3926 (13)	173

Symmetry codes: (i) *x*−1, *y*, *z*; (ii) *x*+1, *y*, *z*; (iii) −*x*+3/2, *y*−1/2, −*z*+3/2; (iv) −*x*+1/2, *y*+1/2, −*z*+3/2.

Table S4. Hydrogen-bond geometry in the structure of **BPD**. (Å, º)

D—H···*A*	*D*—H	H···*A*	*D*···*A*	*D*—H···*A*
C2—H2···N2^{i}	0.95	2.48	3.412 (5)	166
C3—H3···N1^{ii}	0.95	2.58	3.482 (5)	159

Symmetry codes: (i) *x*−1/2, −*y*+3/2, *z*−1/2; (ii) *x*+1, *y*, *z*+1.

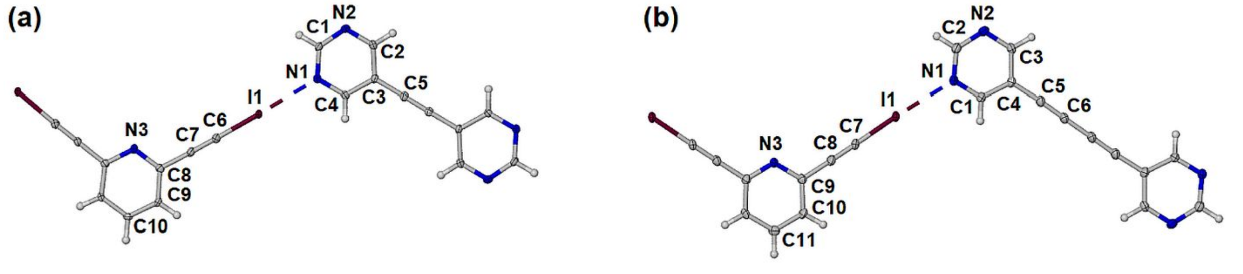

Figure S2. Labelled asymmetric unit of (a) **26DIP•BPE** and (b) **26DIP•BPD** with halogen bonds shown as dashed lines.

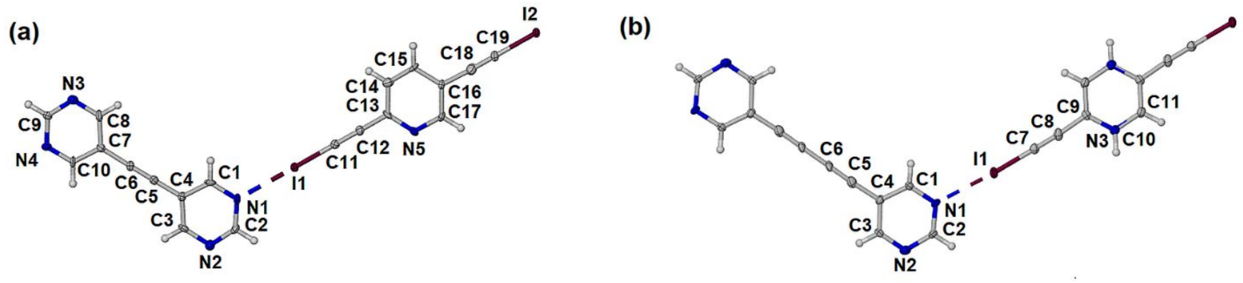

Figure S3. Labelled asymmetric unit of (a) **25DIP•BPE** and (b) **25DIP•BPD** with halogen bonds shown as dashed lines.

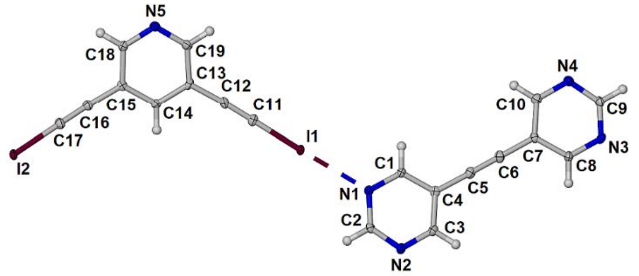

Figure S4. Labelled asymmetric unit of **35DIP•BPE.**

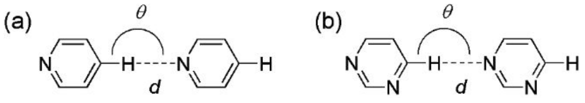

Figure S5. Criteria for CCDC database searches H---N distances. d less than or equal to the sum of the van der Waals radii and θ between 135 and 180º.

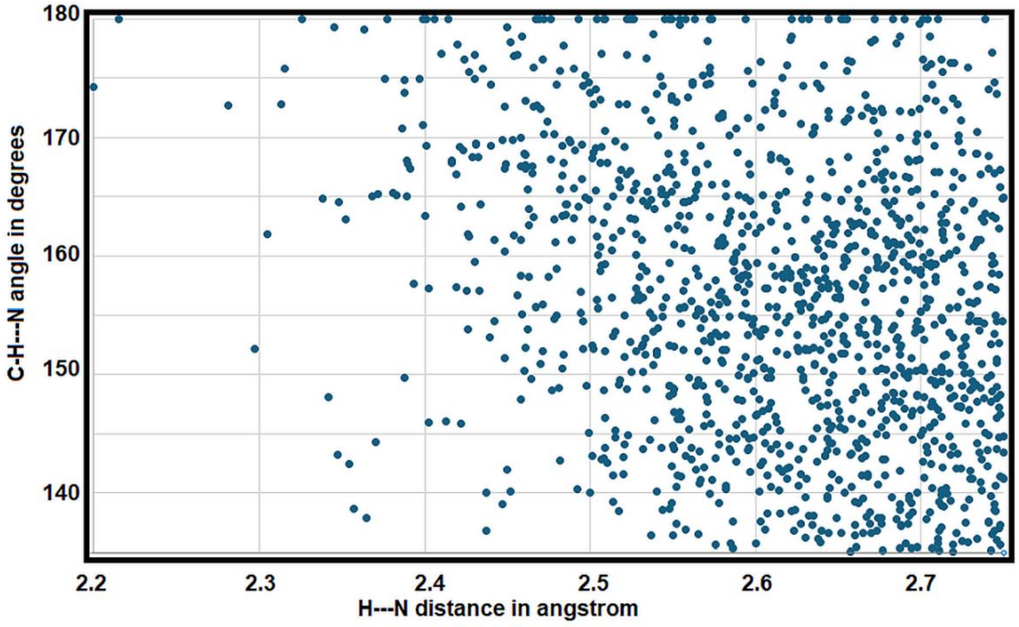

Figure S6. Scattergram plot obtained from the CCDC database search for pyridine-pyridine C-H---N hydrogen bonds in pyridine structures using criteria in Figure S5 (a).

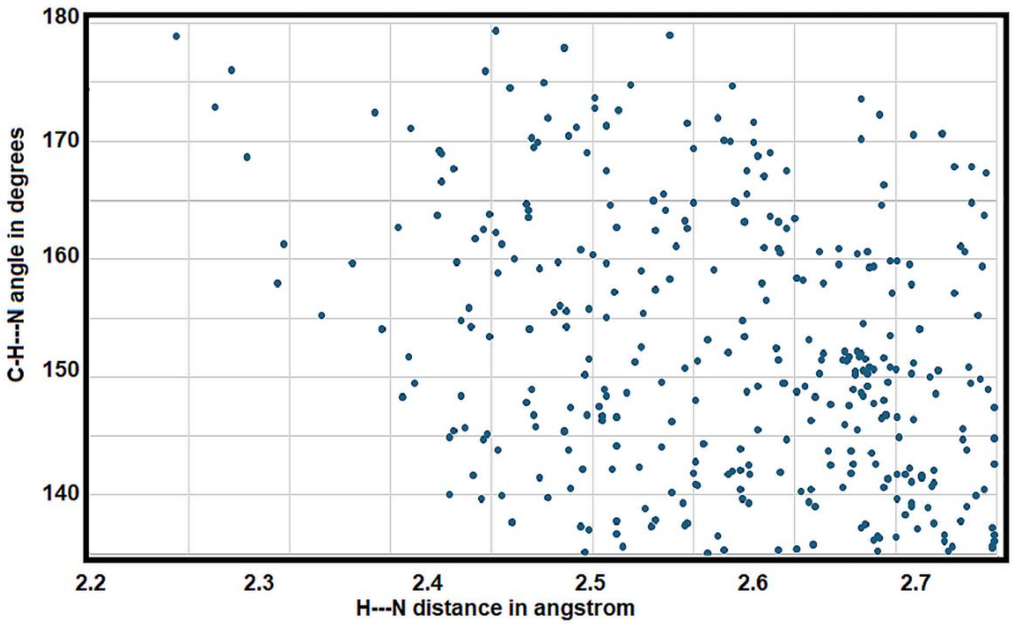

Figure S7. Scattergram plot obtained from the CCDC database search for C-H---N hydrogen bonds in pyrimidine structures using criteria in Figure S5 (b).

Table S5. C-H---I and C-H---π separations in the 5 cocrystals reported here.

Cocrystal	**26DIP•BPE**	**26DIP•BPD**	**25DIP•BPE**	**25DIP•BPD**	**35DIP•BPE**
Pyridine					
C-H---I, Å	3.454(3)	3.444(3)	3.183(10), 3.239(10)	3.335(7)	3.774(3), 3.591(3)
Pyrimidine					
C-H---I, Å	3.102(3)	3.296(3)	3.107(8), 3.121(7)	3.360(7)	3.197(3), 3.276(4)
Pyrimidine-pyrimidine					
C-H---C, Å	-	3.104(4)	-	3.228(10)	-
